# Diet-induced prediabetes: effects of exercise treatment on risk factors for cardiovascular complications

**DOI:** 10.1186/s12986-021-00573-0

**Published:** 2021-04-22

**Authors:** Mluleki Luvuno, Andile Khathi, Musa V. Mabandla

**Affiliations:** Schools of Laboratory Medicine and Medical Sciences, College of Health Sciences, University of KwaZulu-Natal, Private Bag X54001, Durban, South Africa

**Keywords:** Unhealthy diet, Prediabetes, Cardiovascular complications, Exercise intervention, Oxidative stress, Inflammation

## Abstract

**Background:**

An animal model of prediabetes that has been developed in our laboratory using a high fat high carbohydrate diet and lack of physical activity displays risk factors for cardiovascular complications. The effect of exercise against these risk factors in this animal model remains unknown. Therefore, we evaluated the effect of intermittent and regular exercise treatment on the risk factors for cardiovascular complications in this animal model of prediabetes.

**Methods:**

Following prediabetes induction, animals were randomly assigned to the following groups (n = 6): non-diabetic, prediabetic, intermittently exercising prediabetic and regularly exercising prediabetic. Exercise exposure was 7 weeks long. Body weight changes, caloric intake, blood glucose, total cholesterol, and triglyceride concentration was measured after 20 and 29 weeks while blood pressure was only measured after 29 weeks. Plasma endothelial nitric oxide synthase, malonaldehyde, glutathione peroxidase, tumour necrosis factor-alpha and C-reactive protein concentration from the heart were measured 2 weeks post-exercise termination (week 30).

**Results:**

We found increased body weight, caloric intake and mean arterial pressure in the prediabetic group by comparison to the non-prediabetic group. The same trend was observed in blood glucose and triglyceride concentrations. However, all of these parameters were reduced in the intermittently exercising prediabetic and regularly exercising prediabetic groups. This reduction was further accompanied by a decrease in the endothelial nitric oxide synthase, tumour necrosis factor-alpha and C-reactive protein concentration with improved oxidative stress biomarkers.

**Conclusions:**

The progression of pre-diabetes to diabetes is slowed or possibly stopped by exercise (regular or intermittent). Additionally, biomarker profiles indicative of cardiovascular disease in pre-diabetics are improved by exercise.

## Introduction

The abundance of Western-style diets and decreased physical activity in the modern world has resulted in increased incidence of prediabetes and many cases of undiagnosed diabetes [1–4]. This consequently precipitates cardiovascular complications which are more life-threatening than other diabetes complications [1, 5–10]. In our laboratory, an animal model of prediabetes has been developed using an unhealthy high-fat high-carbohydrate diet and lack of physical activity. This animal model opens new avenues for developing different therapeutic approaches in the treatment of this condition [11]. This animal model displays some of the risk factors that are associated with the pathophysiology and pathogenesis of cardiovascular complications in human diabetes mellitus [8, 12–14]. Lipid abnormalities in the concentration of triglycerides, high-density lipoprotein cholesterol (HDL-C) and low-density lipoprotein cholesterol (LDL-C); increased markers of oxidative stress and inflammation as well as high blood pressure were found in this animal model [15–18]. These factors are highly atherogenic and predictive of cardiovascular complications in humans [19, 20]. This shows how this animal model closely represents the human condition.

Due to the abundance of highly palatable foods and the environment’s hedonic food cues, changing dietary habits seems challenging [21–26]. For this reason, in almost all the treatments that are combined with dietary intervention, people tend to relapse back to unhealthy and hedonic eating [21]. Additionally, pharmacological treatment comes with high costs and a need to adhere to dosage instructions [27]. Some of the advantages of exercise therapy include an exercise regimen customized according to the patient’s needs and multi-targeted exercise benefits in the overall health of a patient. Neurobiological studies have shown that exercise is rewarding [28–30]. This suggests that exercise may get pleasurable and tolerable as one continues with it. Therefore, this study sought to evaluate the effects of both regular and intermittent exercise treatment on the changes associated with cardiovascular complications in a high-fat high-carbohydrate diet-induced animal model of prediabetes. Accordingly, we looked at body weight gain, caloric intake, blood glucose and triglyceride concentration as well as mean arterial pressure. Furthermore, we looked at oxidative stress markers: malonaldehyde (MDA), glutathione peroxidase (GPx1); inflammatory markers: tumour necrosis factor-alpha (TNF-α) and C-reactive protein as well as endothelial nitric oxide synthase (eNOS).

## Materials and methods

### Chemicals

All chemicals and reagents were of analytical grade and were purchased from standard commercial suppliers.

### Animals

**Fig. 1:**
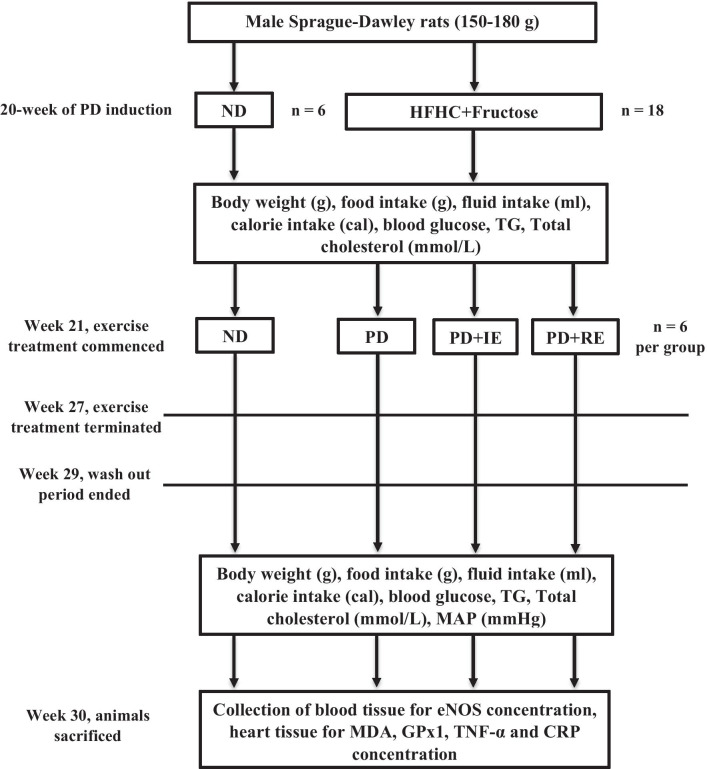
A diagrammatical depiction of the experimental design

Male Sprague–Dawley rats (150–180 g) bred and housed in the Biomedical Research Unit of the University of KwaZulu-Natal under standard laboratory conditions were used in the study. The animals were allowed access to food and fluids ad libitum. All animal experimentation was approved by the Animal Research Ethics Committee of the University of KwaZulu-Natal (Ethical clearance number: AREC/060/017D). Prediabetes was induced in experimental animals by continuously exposing them to a high-fat high-carbohydrate diet supplemented with 15% fructose for 20 weeks (see Fig. 1) [11]. Meanwhile, the non-diabetic animals were given standard rat chow and water during the same 20-week period. At the end of week 20, the animals were randomly assigned to the following groups (n = 6 per group): non-diabetic (ND), prediabetic (PD), intermittently exercising PD (PD + IE) and regularly exercising PD (PD + RE). The animals stayed on the same diet after prediabetes induction until the end of the experiment at week 30. Procedures involving animal care were conducted in conformity with the institutional guidelines for animal care of the University of KwaZulu-Natal.

### Exercise treadmill running protocol

The exercising animals were subjected to moderate endurance exercise on a rat treadmill apparatus consisting of a 2-lane animal exerciser for a 7-week exercise protocol. The first 2 weeks were used for treadmill running acclimatization. During this period, the animals were familiarized with the treadmill apparatus by placing them on the moving treadmill every 3rd day before the actual treadmill running protocol. The duration of the training sessions during the acclimatization period was gradually increased from 5 min to a maximum of 15 min at a constant running speed of 16 m/min. Thereafter, the animals were subjected to either an intermittent or regular treadmill running regimen for 5 weeks [31]. Both exercise regimens were 15 min in duration and continuous but divided into three sessions of 5 min each with a 1 min rest period in between to prevent fatigue. The intermittent treadmill running regimen was performed every other 3rd day. For the PD + RE group, a program of regular treadmill running regimen was performed every 24 h at the same time for 5 weeks. The initial running speed for both intermittent and regular treadmill running regimen was set at 18 m/min and increased by 2 m/min every week for 5 weeks to a maximum of 26 m/min after 5 weeks. Necessary precautionary measures were taken to prevent injuries while constant surveillance of animals was done under the supervision of the Biomedical Research Unit personnel.

### Experimental protocol

Treadmill exercise was carried out for 7 weeks starting on week 21 (see Fig. 1). The protocol duration for the ND and PD groups was the same as the other groups except that they had no access to a treadmill. Body weights, caloric intake, blood pressure, blood glucose, total cholesterol and triglyceride concentrations were measured 1 week pre-exercise (week 20) and one week after termination of exercise (week 29) [32]. Blood pressure was monitored on week 29 using the tail-cuff method. Animals were sacrificed on the 1st day of week 30 to collect blood samples for eNOS measurement. The heart was harvested for MDA, GPx1, TNF-α and C-reactive protein measurements.

### Blood collection and tissue harvesting

For blood collection and heart harvesting, all animals were anaesthetised with Isofor (100 mg/kg) (Safeline Pharmaceuticals (Pty) Ltd, Roodeport, South Africa) via a gas anaesthetic chamber (Biomedical Resource Unit, University of KwaZulu-Natal, Durban, South Africa) for 3 min. Blood was collected by cardiac puncture and then transferred into individual pre-cooled heparinized containers. The blood was then spun in a refrigerated centrifuge (Eppendorf centrifuge 5403, Germany) at 4 °C, 1000× *g* for 15 min. Plasma was collected and stored at − 80 °C in a Bio Ultra freezer (Snijders Scientific, Holland) until ready for biochemical analysis. The heart was removed and stored at -80 °C in a Bio Ultra freezer (Snijders Scientific, Holland) until ready for biochemical analysis.

### Biochemical analysis

GPx1, TNF-α, and C-reactive protein concentration from the heart as well as plasma eNOS were measured using their respective ELISA kits (Elabscience Biotechnology Co., Ltd) according to the manufacturer’s instructions. MDA concentration was measured using an established laboratory protocol [33].

### Analysis of data

All data were expressed as means with standard deviation. Statistical comparisons were performed with GraphPad Prism software (version 5.00, GraphPad Software, Inc., San Diego, California, USA) using one-way analysis of variance (ANOVA) followed by the Tukey–Kramer post hoc multiple comparisons test. A value of *p* < 0.05 was considered statistically significant.

## Results

### Bodyweight changes, caloric intake, blood glucose, triglyceride and total cholesterol concentration

Body weight changes, caloric intake, blood glucose, triglyceride and total cholesterol concentrations were monitored in the ND, PD, PD + IE and PD + RE groups (n = 6, per group) at week 20 and one week after exercise termination. At week 20, there was an increase in body weight gain and total caloric intake in the PD groups (including PD + IE and PD + RE groups) *(ND vs. PD, ND vs. PD + IE and ND vs. PD + RE *p* < 0.05, Table 1). One week after exercise termination, there was a PD effect on weight gain *(ND vs. PD, *p* < 0.05, Table 1), while an exercise effect was present in the PD groups ^#^(PD vs. PD + IE and PD vs. PD + RE, *p* < 0.05, Table 1). A PD effect was present on total caloric intake throughout the experimental period *(ND vs. PD, ND vs. PD + IE and ND vs. PD + RE, *p* < 0.05, Table 1), while an exercise effect was present in the PD groups ^#^(PD vs. PD + IE and PD vs. PD + RE, *p* < 0.05, Table 1). There was a PD effect on blood glucose concentration throughout the experimental period *(ND vs. PD, ND vs. PD + IE and ND vs. PD + RE, *p* < 0.05, Table 1). However, there was an exercise effect in the PD groups ^#^(PD vs PD + IE and PD vs. PD + RE, *p* < 0.05, Table 1). There was a PD effect on triglyceride concentration throughout the experimental period *(ND vs. PD, ND vs. PD + IE and ND vs. PD + RE, *p* < 0.05, Table 1). An exercise effect was also present in the PD groups ^#^(PD vs. PD + IE and PD vs. PD + RE, *p* < 0.05, Table 1).

**Table 1:** Body weight, caloric intake, blood glucose, triglyceride and total cholesterol concentrations in the ND, PD, PD + IE and PDM + RE groups (n = 6, per group) following exercise

Animal groups (n = 6)	Body weight (g)	Total caloric intake (Cal)	Blood glucose (mmol/L)	Triglycerides (mmol/L)	Total cholesterol (mmol/L	
20th week of the prediabetes induction period						
ND	499.70 ± 11.06	223.30 ± 21.92	4.60 ± 0.79	1.49 ± 0.38	3.85 ± 0.23	
PD	566.30 ± 6.51*	311.94 ± 32.02*	6.83 ± 0.25*	3.08 ± 0.27*	4.15 ± 0.26	
PD + IE	564.60 ± 8.62*	308.18 ± 22.65*	6.77 ± 0.38*	3.10 ± 0.15*	4.13 ± 0.31	
PD + RE	567.00 ± 5.90*	310.35 ± 28.37*	6.83 ± 0.30*	3.08 ± 0.18*	4.14 ± 0.17	
The 2nd week after exercise termination						
ND	503.00 ± 18.92	216.30 ± 29.13	4.80 ± 0.10	1.38 ± 0.38		3.87 ± 1.24
PD	589.60 ± 39.02*	339.97 ± 18.09*	7.07 ± 0.40*	5.88 ± 1.49*		4.26 ± 0.72
PD + IE	518.80 ± 65.08^#^	268.93 ± 27.45*^#^	6.37 ± 0.21*^#^	2.58 ± 0.59*^#^		3.93 ± 1.14
PD + RE	505.40 ± 49.77^#^	277.55 ± 10.02*^#^	5.80 ± 0.26*^#^	3.06 ± 0.46*^#^		3.90 ± 1.19

Values are presented as means ± SD. *p < 0.05 denotes comparison with ND group; ^#^*p* < 0.05 denotes comparison with PD group

### Mean arterial pressure and eNOS concentration

**Fig. 2:**
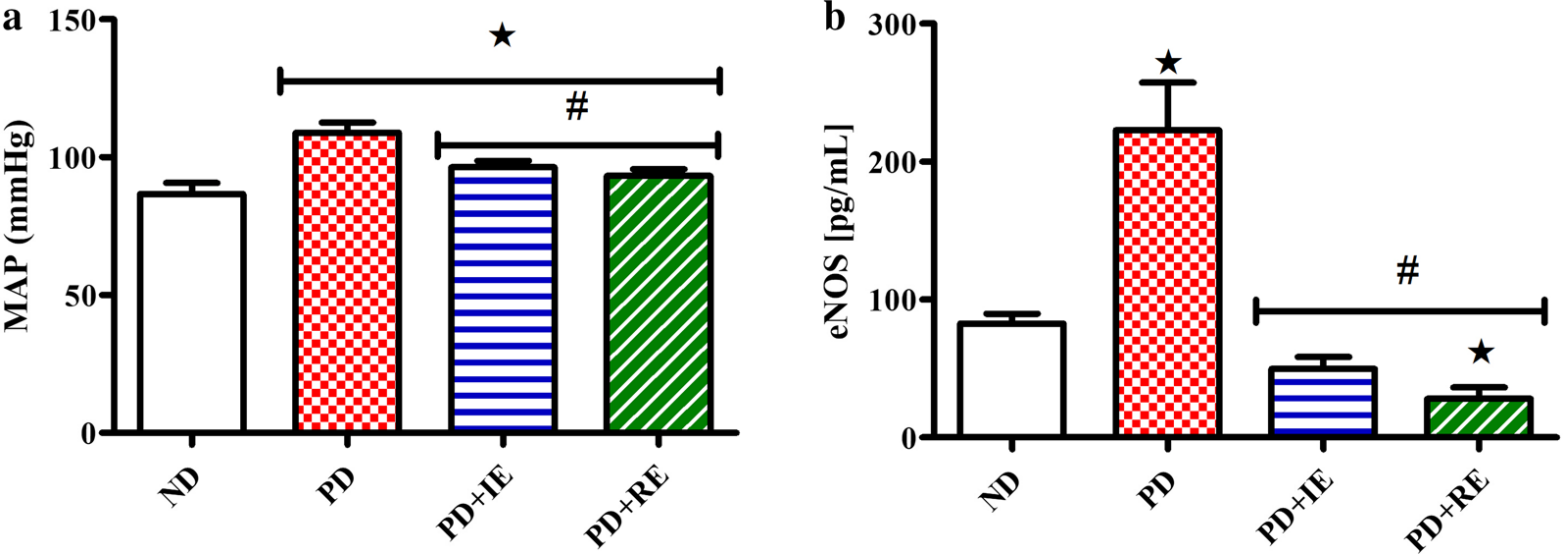
Mean arterial pressure one week after exercise termination (**a**) and plasma eNOS concentration two weeks after exercise termination (**b**) in the ND, PD, PD + IE and PDM + RE groups (n = 6, per group). Values are presented as means ± SD. *p < 0.05 denotes comparison with ND group; ^**#**^*p* < 0.05 denotes comparison with PD group

Mean arterial pressure one week after exercise termination and plasma eNOS concentration two weeks after exercise termination in the ND, PD, PD + IE and PD + RE animal groups (n = 6, per group) were measured. A PD effect was present on mean arterial pressure *(ND vs. PD, ND vs. PD + IE and ND vs. PD + RE, *p* < 0.05, Fig. 2a), while an exercise effect was present in the PD groups ^#^(PD vs. PD + IE and PD vs. PD + RE, *p* < 0.05, Fig. 2a). A PD effect was present in the non-exercising group on eNOS concentration *(ND vs. PD, PD vs. PD + IE and PD vs. PD + RE, *p* < 0.05, Fig. 2b), while regular exercise lowered eNOS concentration ^#^(ND vs. PD + RE, *p* < 0.05, Fig. 2b).

### MDA and GPx1 concentration

**Fig. 3:**
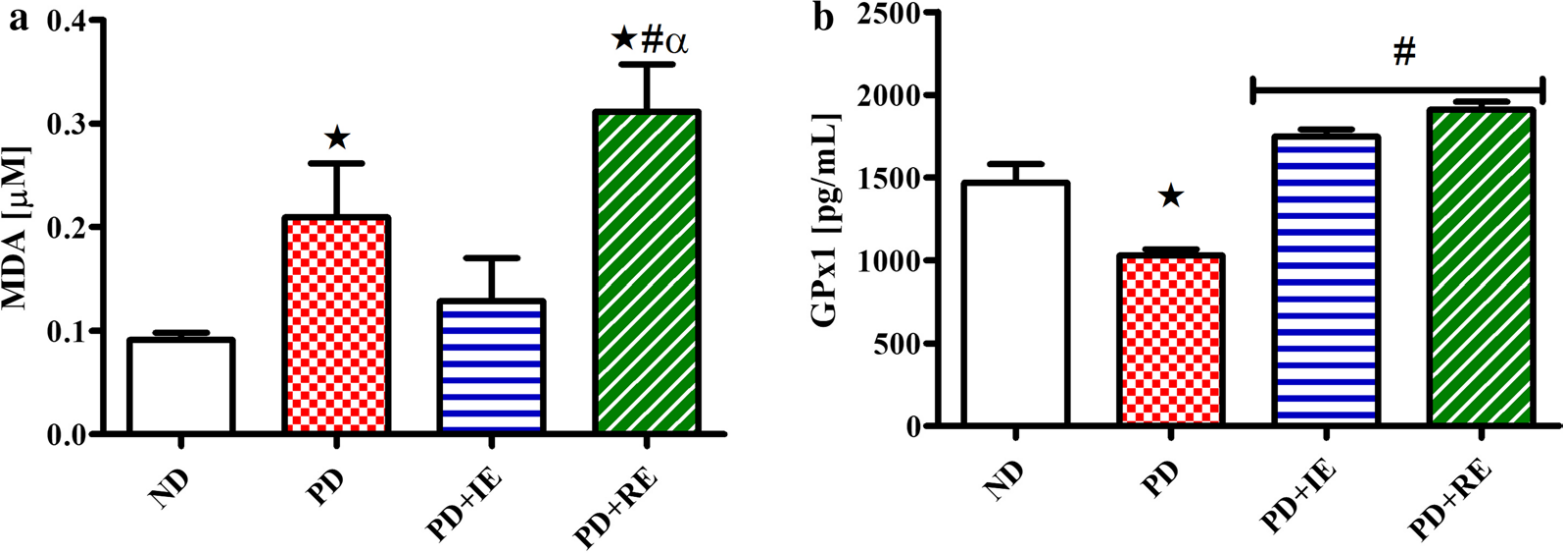
MDA (**a**) and GPx1 (**b**) concentration in the heart tissue of ND, PD, PD + IE and PDM + RE groups (n = 6, per group) two weeks after exercise termination. Values are presented as means ± SD. *p < 0.05 denotes comparison with ND group; ^**#**^*p* < 0.05 denotes comparison with PD group; ^α^*p* < 0.05 denotes comparison with PD + IE group

The concentration of MDA and GPx1 in the heart tissue of ND, PD, PD + IE and PD + RE animal groups (n = 6, per group) two weeks after exercise termination was measured. There was a PD effect on MDA concentration *(ND vs. PD, *p* < 0.05, Fig. 3a). A similar effect was observed in the PD + RE group *(ND vs. PD + RE, *p* < 0.05, Fig. 3a), while a regular exercise effect was present in the PD groups ^#^(PD vs. PD + RE, *p* < 0.05, Fig. 3a) and ^α^(PD + IE vs. PD + RE, *p* < 0.05, Fig. 3a). A PD effect was also present on GPx1 concentration *(ND vs. PD, *p* < 0.05, Fig. 3b), while an exercise effect was present in the PD groups ^#^(PD vs. PD + IE and PD vs. PD + RE, *p* < 0.05, Fig. 3b).

### TNF-α and CRP concentration

**Fig. 4:**
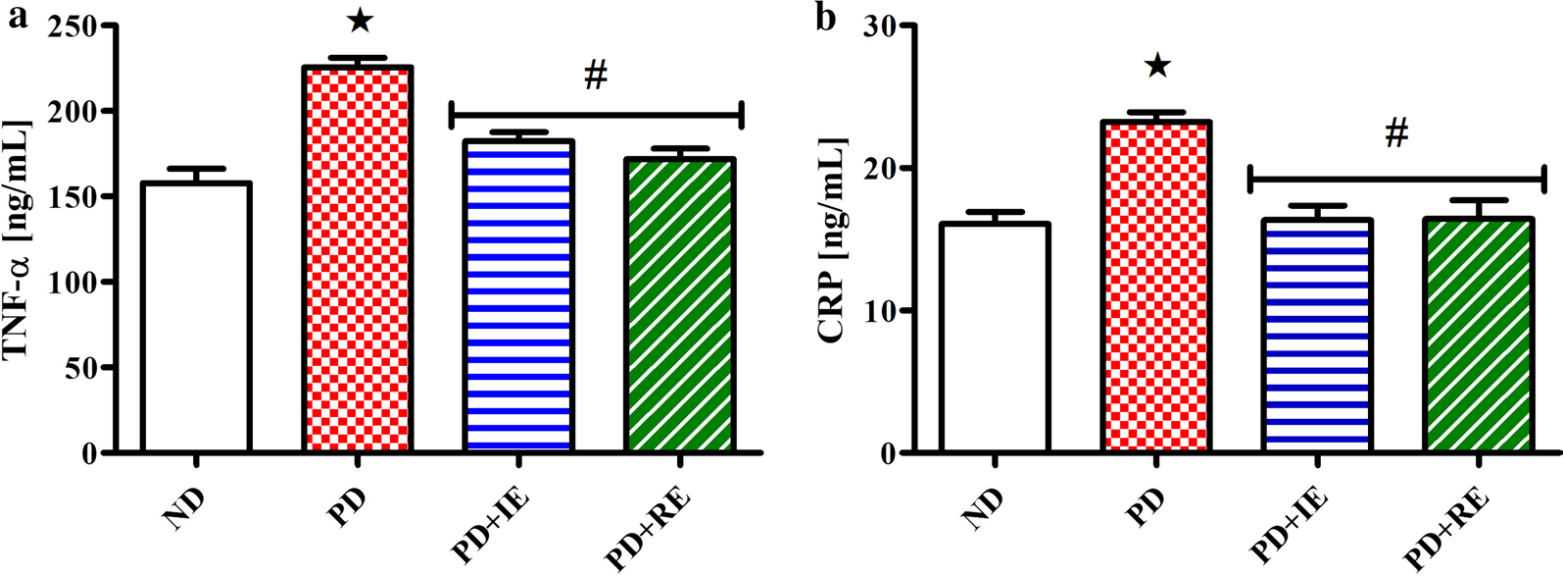
TNF-α (**a**) and CRP (**b**) concentration in the heart tissue of ND, PD, PD + IE and PDM + RE groups (n = 6, per group) two weeks after exercise termination. Values are presented as means ± SD. *p < 0.05 denotes comparison with ND group; ^#^*p* < 0.05 denotes comparison with PD group

TNF-α and CRP concentration in the heart tissue of ND, PD, PD + IE and PD + RE animal groups (n = 6, per group) two weeks after exercise termination were measured. A PD effect was present in the non-exercising group on TNF-α concentration *(ND vs. PD, *p* < 0.05, Fig. 4a) and ^#^(PD vs. PD + IE and PD vs. PD + RE, *p* < 0.05, Fig. 4a). This effect was also present on CRP concentration *(ND vs. PD, *p* < 0.05, Fig. 4b) and ^#^(PD vs. PD + IE and PD vs. PD + RE, *p* < 0.05, Fig. 4b).

## Discussion

The observed lower caloric intake in the exercised prediabetes animals was accompanied by weight loss by comparison to the non-exercised prediabetic animals. We have previously shown that prediabetic animals had reduced insulin sensitivity, increased insulin and ghrelin concentrations as well as decreased leptin concentrations [11]. Consequently, this resulted in increased feeding and caloric intake. Therefore, a lower caloric intake in the exercised prediabetic animals after exercise could suggest that exercise assisted in the restoration of the homeostatic relationship between insulin, ghrelin, and leptin. This could explain the adjustments in caloric intake and weight gain in the exercised prediabetes animals. This agrees with other literature which shows that exercise has a positive effect on food intake, appetite hormones and weight management [34–40]. Furthermore, studies have shown that lower caloric intake may have a positive effect on the physiological markers associated with prediabetes [41–43].

Blood glucose concentrations were lowered in the exercising prediabetic animals at one-week post-exercise termination. Furthermore, the exercising animals were found to have reduced insulin concentrations suggesting that exercise may have improved insulin sensitivity [11, 44]. The lowered blood glucose concentration observed in the exercising prediabetes animals may be due to increased GLUT4 translocation via the action of insulin [45, 46]. Furthermore, improved insulin sensitivity may have enhanced carbohydrate loading in the exercising prediabetes animals thus shunting some glucose towards glycogen storage in the muscle and liver in preparation for the next exercise training [47].

There were no total cholesterol concentration changes in the groups which may be because our animals were still prediabetic and had not developed full-blown diabetes. We have previously shown that high triglyceride concentration is accompanied by high blood glucose concentration and reduced insulin sensitivity in pre-diabetic animals [11]. This triglyceride concentration was lowered in the exercising prediabetes animals in the present study. This may suggest that exercise leads to enhanced lipid metabolism and insulin sensitivity thus decreasing plasma glucose concentration [48].

We found that when blood glucose and triglyceride concentration were lowered in the exercising prediabetes animals one-week post-exercise termination there was a concomitant improvement in the mean arterial pressure. One can easily assume that abnormalities associated with increased blood pressure were also attenuated in these animals. However, we found higher plasma eNOS concentration in the non-exercising prediabetes animals in the presence of high mean arterial pressure when compared to the non-diabetic. This high eNOS concentration that co-exists with high mean arterial pressure in the non-exercising prediabetic animals might be a compensatory response for insufficient NO following prolonged ingestion of the high-fat high-carbohydrate diet. [49–55]. Two weeks of post-exercise termination, we observed a reduction in the eNOS concentration. This reduction in eNOS concentration in the exercising prediabetic animals was accompanied by improved mean arterial pressure in the animals suggesting the long-lasting beneficial effects of exercise training on NO production.

Studies have shown that hypertension induces responses that elicit oxidative stress and inflammatory responses [56–60]. Indeed, we found increased MDA concentration and decreased GPx1 concentration in the heart tissue of the non-exercising prediabetic animals. However, we found reduced MDA concentrations along with elevated GPx1 concentrations in the intermittent exercising prediabetic animals. Meanwhile, the regular exercising prediabetic animals had elevated MDA concentrations despite increased GPx1 concentration. The reduced MDA concentration with elevated GPx1 concentration in the intermittent exercising animals agrees with other studies which show that an elevation in antioxidants prevents the onset of oxidative stress through suppression of ROS generation [61, 62]. This may lead to an assumption that intermittent exercise was better than regular exercise in alleviating oxidative stress markers in the heart. However, the increase in both MDA and GPx1 concentrations in the regular exercising prediabetic animals can be linked to an adaptational increase in both ROS production and antioxidant enzyme production following exercise. Recent studies have shown that increased ROS generation may not always culminate in oxidative stress and destruction of cellular structures [63, 64]. However, an increase in antioxidant enzyme concentration accompanied by ROS production prevents the development of oxidative stress in the cells and also activates the pathways that regulate growth, differentiation and cell proliferation [64, 65]. Therefore, this may suggest that regular exercise was more beneficial to heart tissue health than intermittent exercise [66–68]. Furthermore, exercise-induced ROS production has been shown to play a required role in muscle adaptation to training [66]. However, the adaptational increase in MDA concentration was only observed in regular exercising animals. This suggests that an exercise-induced ROS generation does not only depend on the intensity and duration of exercise but also the frequency of exercise.

The findings on the biomarkers of inflammation in the present study are of interest because an increase in the marker for lipid peroxidation, MDA, is usually associated with inflammation [69, 70]. However, this correlation was not observed in the present study particularly in the regular exercising prediabetic animals. For the intermittent exercising prediabetic animals, the decrease in MDA concentration with an increase in GPx1 concentration in the heart tissue clearly shows that there was a physiological balance between oxidants and antioxidants in the heart tissue. However, a decrease in TNF-α and C-reactive protein concentrations despite increased MDA and GPx1 concentrations in the heart tissue of the regular exercising animals two weeks after exercise termination reveals that there was no oxidative damage and inflammation in the heart tissue. This suggests that an adaptational increase in the antioxidant enzymes as indicated by an increase in GPx1 concentration following exercise was high enough to suppress the effects of ROS production and prevent oxidative damage and inflammation to occur in the heart tissue of the exercising animals [64, 71–73].

## Conclusion

The progression of pre-diabetes to diabetes is slowed or possibly stopped by exercise (regular or intermittent). Additionally, biomarker profiles indicative of cardiovascular disease in pre-diabetics are improved by exercise.

## Data Availability

The datasets used and/or analyzed during the current study are available from the corresponding author on reasonable request.

## Abbreviations

eNOS: Endothelial nitric oxide synthase
GLUT4: Glucose transporter 4
GPx1: Glutathione peroxidase
PD + IE: Intermittently exercising PD
MDA: Malonaldehyde
NO: Nitric oxide
ND: Non-diabetic
PD: Prediabetic
ROS: Reactive oxygen species
PD + RE: Regularly exercising
TNF-α: Tumour necrosis factor-alpha
